# WIP Remodeling Actin behind the Scenes: How WIP Reshapes Immune and Other Functions

**DOI:** 10.3390/ijms13067629

**Published:** 2012-06-21

**Authors:** Elad Noy, Sophia Fried, Omri Matalon, Mira Barda-Saad

**Affiliations:** The Mina and Everard Goodman Faculty of Life Sciences, Bar-Ilan University, Ramat-Gan 52900, Israel; E-Mails: elad.noy@biu.ac.il (E.N.); sophia.fried@biu.ac.il (S.F.); omri.matalon@live.biu.ac.il (O.M.)

**Keywords:** WIP, WASp, actin, cytoskeleton, immune response, lymphocytes, TCR signaling

## Abstract

Actin polymerization is a fundamental cellular process regulating immune cell functions and the immune response. The Wiskott-Aldrich syndrome protein (WASp) is an actin nucleation promoting factor, which is exclusively expressed in hematopoietic cells, where it plays a key regulatory role in cytoskeletal dynamics. WASp interacting protein (WIP) was first discovered as the binding partner of WASp, through the use of the yeast two hybrid system. WIP was later identified as a chaperone of WASp, necessary for its stability. Mutations occurring at the WASp homology 1 domain (WH1), which serves as the WIP binding site, were found to cause the Wiskott-Aldrich syndrome (WAS) and X-linked thrombocytopenia (XLT). WAS manifests as an immune deficiency characterized by eczema, thrombocytopenia, recurrent infections, and hematopoietic malignancies, demonstrating the importance of WIP for WASp complex formation and for a proper immune response. WIP deficiency was found to lead to different abnormalities in the activity of various lymphocytes, suggesting differential cell-dependent roles for WIP. Additionally, WIP deficiency causes cellular abnormalities not found in WASp-deficient cells, indicating that WIP fulfills roles beyond stabilizing WASp. Indeed, WIP was shown to interact with various binding partners, including the signaling proteins Nck, CrkL and cortactin. Recent studies have demonstrated that WIP also takes part in non immune cellular processes such as cancer invasion and metastasis, in addition to cell subversion by intracellular pathogens. Understanding of numerous functions of WIP can enhance our current understanding of activation and function of immune and other cell types.

## 1. Introduction

Rearrangement of the actin cytoskeleton is a key cellular event important for multiple cell functions, and thus is a highly regulated process [1]. The formation of actin filaments (F-actin) from globular monomeric subunits (G-actin) is at the core of cytoskeletal reorganization processes. Spontaneous actin polymerization is prevented by actin monomer-sequestering proteins, actin severing proteins, as well as by the inherent instability of actin dimers. Thus, actin polymerization is dependent on the initial polymerization of three or more G-actin monomers, acting as a stable nucleus to which additional actin monomers may be assembled. Formation of these actin nuclei is promoted by actin nucleation proteins. The actin nucleation protein complex Arp2/3 works by mimicking two actin subunits, thereby serving as a template for actin assembly [2]. Arp2/3 constitutes a central actin nucleation protein complex, responsible for enabling actin polymerization at an angle from existing actin filaments, thereby enabling branching of the actin network. The activity of Arp2/3 is greatly enhanced by binding to proteins called nucleation-promoting factors (NPFs) [3–6]. Wiskott-Aldrich syndrome protein (WASp) is a cardinal NPF, first discovered as the protein encoded by a gene mutated in patients suffering from Wiskott-Aldrich syndrome (WAS). WAS is an X linked disorder causing a wide spectrum of clinical manifestations including immunodeficiency, recurrent infections, eczema, and susceptibility to the development of autoimmune diseases [7–11]. WAS is a result of mutations occurring in the *WAS* gene, with clinical manifestation dependent on the genotype. The spectrum of clinical severity of WAS includes a milder variant, called X-linked thrombocytopenia (XLT). XLT is mainly caused by missense mutations resulting in expression of defective WASp, commonly in reduced quantity, while WAS, the more severe clinical manifestation is generally caused by a complete absence of WASp [12,13].

WASp is exclusively expressed in hematopoietic cells, while its counterpart, neural-WASp (N-WASp), is ubiquitously expressed [14]. While several different WAS and XLT-causing mutations were identified [15], most of them map to the binding site for WASp-interacting protein (WIP), and some of them were shown to disrupt the interaction of these proteins, thereby promoting WASp degradation [16]. WIP was first discovered as the binding partner of WASp associated with actin polymerization [17], and was later demonstrated to function as a chaperone of WASp [18,19]. Immune responses, mainly those that are dependent on cellular functions that require actin polymerization, are impaired in WAS patients. In lymphocytes, WIP was found to fulfill different roles in B cells, T cells, and natural killer (NK) cells, with WIP deficiency having varying consequences for cellular proliferation, activation and function. Interestingly, a stop codon mutation in WIP was recently found to cause a WAS-like disease, also characterized by WASp degradation [20]. Nevertheless, in addition to the role of WIP in interacting with WASp, WIP regulates actin polymerization, and other cellular processes, in a WASp independent manner through its contact with a variety of other binding partners. Understanding of the WASp independent functions of WIP is in its early stages.

Here, we review the various mechanisms of WIP activity, the importance of WIP for the functioning of immune cells, its newly discovered roles beyond the immune system, its involvement in the development of metastasis, and its subversion by certain intracellular pathogens.

## 2. WIP Family Proteins

Human WIP is a 503 amino acid (aa) proline rich protein with a multidomain structure. It is part of the verprolin (Vrp1p) family, which also includes WIP-CR16 homologous/WIP related (WICH/WIRE) andcorticosteroids and regional expression 16 (CR16) [21]. Verprolin is a yeast actin binding protein that participates in actin organization and is important for cell polarity and endocytosis [22,23]. Importantly, WIP is able to compensate for cytoskeletal defects in verprolin deficient yeast cells [24].

WICH/WIRE was discovered simultaneously by two groups, hence its two names; the protein is expressed in various cell types such as brain, lung, and colon and was shown to participate in receptor-mediated endocytosis [25]. WICH/WIRE binds to WASp and N-WASp [26], and was shown to promote formation of cross linked actin filaments [27] and to translocate to peripheral actin assembly sites following platelet-derived growth factor (PDGF) treatment [28].

The CR16 protein is expressed primarily in the brain, and is also expressed in the heart, lung, and testis [29,30]. In the brain [31] and in human testis cells [32], CR16 was found to bind N-WASp. CR16 deficiency in mice causes male-specific sterility [32,33].

An additional WIP related protein is a 403 aa long [34] truncated isoform of WIP lacking the WASp binding region. This isoform is referred to as mini-WIP, and was found to be expressed in the human peripheral blood, cell lines (EBV transformed B cell lines and the Jurkat T cell line), and mouse splenocytes. The biological importance of mini-WIP is yet to be demonstrated; it would be interesting to learn how this protein participates in actin regulation independently of WASp.

## 3. WIP Structure and Binding Partners

**Figure 1:**
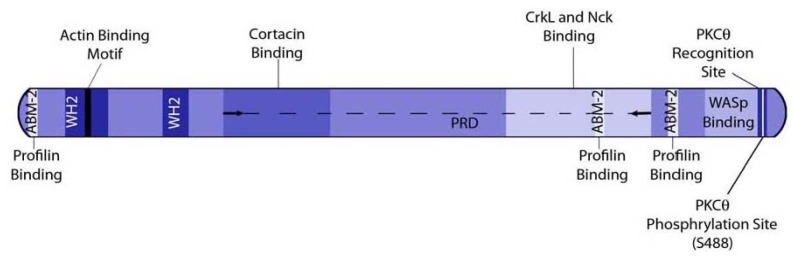
A model of WASp-interacting protein (WIP) structure. Actin based motility 2 (ABM-2), WASP homology 2 (WH2), Proline rich domain (PRD).

WIP is a multidomain protein containing various motifs allowing it to bind to a variety of associated proteins (Figure 1). The *N*-terminal region of WIP is referred to as the verprolin homology region (V-domain), and is present in all verprolin family members. The V-domain consists of residues 1–112 and includes two stretches of high verprolin similarly called WASP homology 2 (WH2) domains. An actin binding consensus sequence is present in the first WH2 of WIP (KLKK, aa 45–48), and was suggested as a potential direct actin binding site of WIP [17]. Indeed, WIP was shown to bind G-actin and to stabilize F-actin [35]. CR16 and WICH/WIRE were also shown to bind both G- and F-actin.

WIP contains three actin based motility 2 (ABM-2) regions, the first of which, comprised of aa 8–13, is located in the V-domain, while the other two (aa 379–384, aa 427–432) [17,36,37] are located closer to the C terminus. ABM-2 regions form potential binding sites for profilin, an actin binding protein that has an important role in the maintenance of the actin pool and the dynamic turnover of the actin cytoskeleton, and was shown to bind to WIP [17]. This interaction may be important for the role of WIP in actin dynamics, and also link profilin to other WIP-binding partners related to actin regulation. The *N*-terminus of WIP also contains a glycine rich region, located at aa 64–107, that may serve as a flexible molecular hinge.

The central region of WIP is rich in proline residues, allowing it to bind Src homology 3 (SH3) containing proteins such as Nck1, CrkL, and cortactin. Nck is an adaptor protein that participates in the signaling cascade leading to actin polymerization in T cells [38–40]. WIP was found to interact with the second SH3 domain of Nck via a stretch of its proline rich domain (PRD), located at aa 321–415 [41]. This region of WIP also mediates the binding of the first SH3 domain of CrkL [42]. The binding of WIP to CrkL is responsible for the recruitment of the CrkL-WIP-WASp complex to the T cell-antigen presenting cell (APC) contact site, termed the immunological synapse (IS), following lymphocyte activation [42]. Cortactin is an NPF that activates Arp2/3, and thus enhances the formation of actin branches and the stabilization of actin filaments [43]. The interaction between WIP and cortactin is mediated by the fourth SH3 domain of cortactin and a stretch of the WIP PRD, located within the region of aa 136–205 [44]. Recently, it was shown that the interaction between WIP and cortactin is essential for the formation and maturation of actin-rich cellular protrusions, secretion of matrix degrading enzymes (matrix metalloproteinases, MMPs), and degradation of extracellular matrix (ECM) by murine dendritic cells [45].

WIP was shown to bind and activate Hck [46], a Src family kinase expressed mainly in hematopoietic cells, which participates in formation of membrane protrusion structures [47], phagocytosis, and monocyte activation [48]. Hck was also found to be crucial for signal transduction events occurring after integrin ligation (integrin outside-in signaling), mediating neutrophil activation and granule secretion [49]. Since this interaction is clearly mediated by the SH3 domain of Hck [46], it is possible that Hck binds to the PRD of WIP.

The WIP *C*-terminal region contains a binding site for WASp/N-WASp (see Figure 1). NMR studies mapped the WASp binding site of WIP to aa 451–485 [50,51], while the WH1 domain of WASp (amino acids 47–137) is thought to contain the binding site for WIP. The interaction between WIP and WASp is crucial for WASp stability, since the lack of WIP or an impaired WIP-WASp interaction results in WASp degradation [16] by the calcium-dependent, non-lysosomal cysteine protease, calpain, or by the proteasome [18,19]. Interestingly, it was shown that N-WASp is susceptible to proteasome degradation and not to calpain [52], and that the stability of N-WASp is not solely dependent on WIP, suggesting that in this case, WIP homologs WICH/WIRE and CR16, which bind to N-WASp [26,31], potentially fulfill the protective role of WIP. WIP was also found to regulate WASp localization and function, since it was shown that WIP conveys WASp to areas of active actin assembly such as the IS [42].

WIP contains a single protein kinase C theta (PKCθ) consensus phosphorylation site (RxxSxR, residues 485–490) [42] in proximity to the WASp binding site. Following T cell antigen receptor (TCR) ligation, WIP is phosphorylated on serine 488 [42]. Previous studies suggested that upon WIP phosphorylation, WASp diassociates from WIP, and it was proposed that this dissociation is required for WASp function. A subsequent study, however, proved WIP-WASp dissociation is not required for normal WASp function [53]. This apparent contradiction was subsequently explained by the fact that the antibody used in the initial study was raised against a WASp adjacent site, possibly causing displacement of WASp from WIP following activation. Using a different monoclonal antibody, no dissociation was observed [34]. However, the role of WIP phosphorylation in the regulation of WIP-WASp complex formation remains to be clarified. The regulation of this complex is very intricate, due to the fact that both WIP and WASp each functionally interact with a multitude of binding partners.

A novel interaction of WIP-WASp homologs was found in drosophila; Blown-fuse protein was shown to compete with WASp for the binding of Sltr (the *Drosophila* ortholog of WIP). This raises the possibility of a human homolog of Blown-fuse, regulating the WIP-WASp complex by competing with WASp for the binding of WIP [54].

## 4. Roles of WIP in Immune Cells

As previously described, WAS, caused by reduced expression or enhanced degradation of WASp, is characterized by recurrent infections, thrombocytopenia, and a susceptibility to malignancies and autoimmune disorders [7]. WIP deficiency, as shown in WIP^−/−^ mice, causes hematological and immunological abnormalities beyond those encountered in WASp^−/−^ cells [55]. WIP^−/−^ mice exhibit severe lymphopenia, inclination to inflammatory diseases, such as ulcerative colitis, and hypersensitive pneumonitis. Interestingly, in contrast to WASp^−/−^ mice [56], platelet volume is normal and no thrombocytopenia is observed. Additionally, WIP^−/−^ mice die prematurely, between the 16th to the 40th week of age. Taken together, these findings highlight the essential role of WIP in immune cells [55].

### 4.1. T Lymphocytes

T lymphocytes, belonging to the adaptive arm of the immune system, are the chief regulators of the immune response [57]. T cells are responsible for recognizing antigens displayed by APCs, eliciting an appropriate immune response. Additionally, a subset of T cells, called cytotoxic T cells, is responsible for killing virally infected or tumorally transformed cells.

**Figure 2:**
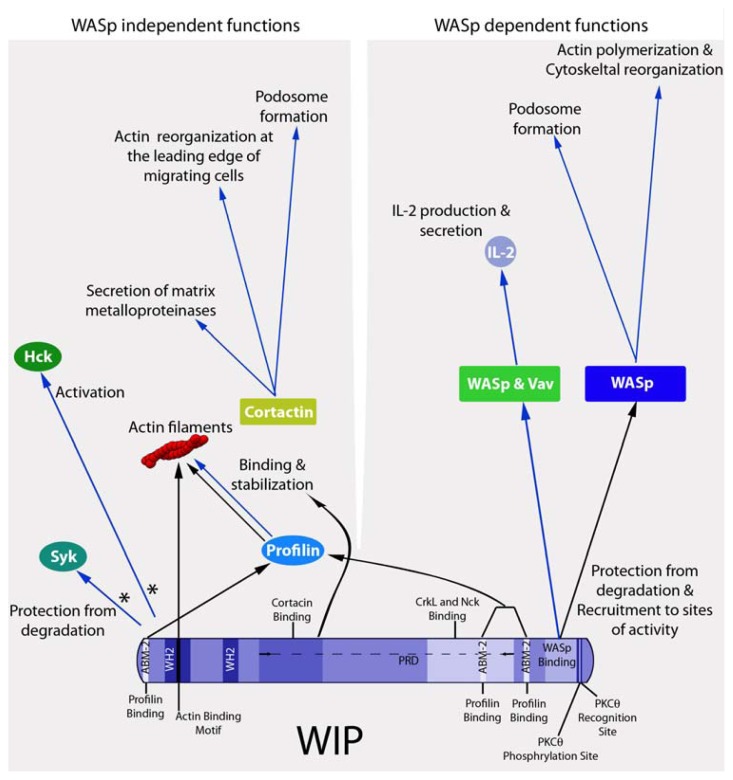
WIP interaction with its various binding partners, cooperating toward cellular functions. WIP interacts with various binding partners involved in multiple cellular processes, including actin polymerization, cytoskeletal rearrangement, migration and cellular activation. WIP was originally identified as a chaperone of WASp, protecting it from degradation and allowing it to promote actin polymerization and the rearrangement of the actin cytoskeleton. WIP is additionally responsible for recruiting WASp to its sites of activity. WASp and WIP also cooperate with Vav, inducing IL-2 production in activated T cells (**right**); In addition to the WASp-dependent functions of WIP, some roles of WIP rely on interactions with additional proteins. The PRD of WIP binds to cortactin, promoting actin reorganization, podosome formation and maturation, cellular migration and the secretion of matrix metalloproteinases. WIP also enhances actin stability by directly binding to actin filaments and to the actin sequestering protein, profilin. Other important binding partners of WIP are Hck, a Src family kinase involved in phagocytosis and monocyte activation, and Syk, a protein involved in mast cell signaling, protected from degradation by WIP (**left**). Black arrows indicate protein binding to WIP while blue arrows represent WIP dependent function. * The role of WASp in the interaction between WIP and these proteins has not yet been fully defined.

T lymphocytes of WAS patients display abnormal morphology, and defects in actin rearrangement, proliferation and cell spreading in response to TCR stimulation [58–60]. WASp deficient mice exhibit a substantial decrease of peripheral blood T cell numbers, aberrant TCR induced proliferation and reduced IL-2 production [56,61,62]. WIP protects WASp from cellular degradation by calpain and the proteasome [19]; most WAS-associated mutations disrupt the interaction between WIP and WASp, leading to enhanced WASp degradation. In order to address the specific roles of WIP in the immune system, WIP^−/−^ mice were generated. T cells from WIP^−/−^ mice show highly disrupted cellular function [63]. Following TCR ligation, these cells fail to secrete IL-2, proliferate, or to increase their F-actin content. Using the T cell spreading assay over an anti-CD3 surface [64–66], it was shown that their spreading, polarization and protrusion formation are highly defective [63]. WIP was found to cooperate with signaling proteins such as Vav1, a guanine exchange factor that regulates NF-AT/AP1-mediated gene transcription in T cells [67]. WIP and Vav1 cooperate to enhance IL-2 production in a WASp-dependent manner [68] (see Figure 2). Another WIP-WASp dependent mechanism enhancing IL-2 production in T cells involves Fyn. In activated T cells, Fyn binds to the WASp *N*-terminus, and a trimeric complex of WIP-WASp-Fyn is formed. This complex was shown to play an important role in IL-2 production mediated by TCR signaling [69].

Importantly, WASp and WIP double knockout cells fail to proliferate in response to soluble and immobilized anti-CD3, while WASp deficient cells proliferate normally in the presence of soluble anti-CD3 presented by APCs [70]. Moreover, the IL-2 responsiveness of double knockout T cells is defective. In response to IL-2, these double knockout T cells exhibit defective IL-2R signaling, as they fail to phosphorylate the signal transducer and activator of transcription 5 (STAT5) and induce the expression of STAT5 dependent genes, also failing to upregulate CD25 [70].

Several studies have reported that WASp deficient T cells exhibit aberrant actin responses and spreading following TCR activation [61,71,72]. Nevertheless, others showed that WASp-deficient T cells can form stable conjugates with APCs, form IS, and polymerize actin normally [62]. In WIP^−/−^ T cells, on the other hand, IS formation is severely impaired. This can be explained by the fact that while WASp is required for IS stability and reformation after periodic breaks, it is not required for initial IS formation [73]. Interestingly, it has been shown that WASp and PKCθ have opposing roles regarding IS stability. PKCθ destabilizes the IS by breaking its symmetry structure and reinstating T cell migration while WASp is essential for IS reformation and stabilization. WIP regulates WASp activity and in turn, is phosphorylated by PKCθ. As PKCθ has an opposing role to that of WASp in determining IS stability, this suggests an intriguing role for WIP phosphorylation in the balance of IS formation, stabilization and destabilization. Another study examined the migration of T cells in response to the chemokine SDF-1α. It was found that while chemotaxis of WASP^−/−^ cells was only mildly reduced, in both WIP^−/−^ and double knockout cells, chemotaxis was severely impaired [74].

Taken together, these data indicate that the role of WIP in T cell function goes beyond simply protecting WASp from degradation. The multiple WASp independent roles of WIP can be explained by its ability to interact directly with actin and actin regulatory proteins.

### 4.2. B Lymphocytes

B lymphocytes comprise the second arm of adaptive immunity, eliciting both antibody-mediated responses and mediating T cell priming as specialized APCs. B-cell activation via B-cell receptor (BCR) ligation with antigen leads to B-cell activation, proliferation and differentiation [75].

WIP^−/−^ mice exhibit hematological and immunological abnormalities including a substantial reduction of B cells [55]. In contrast to T cells, B cells from WIP^−/−^ mice exhibit an enhanced overall response to non-specific stimuli such as LPS and anti-CD40 mAb, with or without IL-4. These cells exhibit increased proliferation, high levels of CD69, and increased IL-2R expression. As the internalization of the BCR terminates the signaling process responsible for B cell activation, it was suggested that defective BCR internalization might strengthen B cell activation [63]. However, the BCR internalization process is normal in WIP^−/−^ cells [63]; thus, the enhanced response of these cells to BCR ligation is yet to be explained. Interestingly, a recent study demonstrated that WASp deficient B cells are only mildly hyper-responsive to activating signals and display reduced BCR internalization [76]. These findings yet again support the existence of WASp-independent mechanisms for WIP functions in immune cells.

### 4.3. NK Cells

#### 4.3.1. WIP-WASp Complex Dynamics Following NK Activation

Natural killer (NK) cells are lymphocytes belonging to the innate immune system, specialized for elimination of transformed and virally infected cells. NK cells function via direct lysis of target cells and the production and release of chemokines and cytokines, further assisting in the regulation of immune responses. In NK cells, it was shown that WASp binding to WIP is constitutive and independent of the activation state of the cells [77]. Following NK cell conjugation and activation, WASp is recruited to the natural killer immunological synapse (NKIS) and forms a 1,300 kDa multiprotein complex, which consists of WIP, actin, and myosin IIA [77]. The cellular activation of NK cells results in WIP phosphorylation by PKCθ, in correlation with formation of the complex. This phosphorylation does not interrupt the association of WASp with WIP in NK cells [77].

The WIP-WASp complex is not affected as part of the process of cellular inhibition via the inhibitory receptor KIR2DL1, while the recruitment of actin and myosin IIA to the complex is abolished. The distribution of WIP in resting cells is cytoplasmic, while following NK cell conjugation and activation, the WIP-WASp complex is recruited to activating NKIS at the membrane, and is colocalized with F-actin and perforin [78]. It was shown that the assembly of the multiprotein complex of WASp, WIP, actin and myosinIIA is not WASp dependent, since upon activation, a mutated WIP that cannot bind WASp successfully recruits myosin IIA and actin [77].

#### 4.3.2. WIP and WASp Have Differential Activities in NK Cells

A human WIP deficiency, caused by a stop codon mutation in the WIP coding gene, causes an immunodeficiency disease with similar characteristics to those of WAS, including an undetectable level of WASp expression. It was found that the number of NK cells (and their abundance among the various blood cell populations) is increased in WIP-deficient patients, while NK cell functional activity and cytotoxicity were found to be drastically reduced [20].

The role of WIP in NK cell cytotoxicity appears to be crucial, as WIP knockdown results in a significant reduction of NK-mediated cytotoxicity, and WIP overexpression enhances it [77,79]. In addition, WIP colocalizes with lytic granules in both resting and activated NK cells, a function that was shown to be independent of WASp. Following NK conjugation and WIP activation, the lytic granules are colocalized and polarize towards the NKIS. WIP is crucial for lytic granule polarization, since knockdown of WIP inhibits this process. On the other hand, WIP knockdown impairs the conjugation of NK cells to the target cells [79], as caused by WASp deficiency, indicating that WIP has both WASp dependent and independent functions in the regulation of NK cell activity.

NK cells of WAS patients exhibit a complex pathology, and a few cases of NK cell mosaicism including WASp negative and WASp positive subpopulations were reported [80–82]. The presence of NK subpopulations differing in their WASp expression and in their occurrence in the blood can be explained by somatic reversions of WAS-causing mutations, and potentially by mutations or second-site mutations, mitigating the effect of the original mutation [80]. In some of these cases, an increased percentage of WASp positive NK cells were observed in the patient’s blood, suggesting a selective advantage for this subpopulation [80,81]. Interestingly, similarly to WIP-deficient patients, WAS patients show normal or elevated NK cell percentages [20,78,83]; however, NK cells from these patients exhibit defective NK cell cytotoxicity [78,83–85]. The presence of an increased or normal relative number of NK cells in WAS patients, together with some of the cases exhibiting mosaicism, in which no selective advantage for WASp positive NK cells was detected, raises the question of the exact role of the WIP-WASp complex in NK cell development and proliferation.

### 4.4. Myeloid Cells

WIP plays a cardinal role in the migration of the phagocytes, macrophages and dendritic cells. These cells migrate using actin-rich cellular protrusions called podosomes. A podosome is a short lived structure, containing a core of WASp, WIP, Arp2/3 and actin, surrounded by adaptor molecules (including talin and vinculin), which link the podosome to integrin molecules [86–88]. Macrophages and dendritic cells isolated from WAS patients lack podosomes entirely, as do WIP^−/−^ dendritic cells [37,89]. In addition to protecting WASp from degradation, WIP was also found to regulate the localization of WASp to podosomes [18]. Interestingly, WIP^−/−^ cells form adhesion structures named “large focal contacts” rather than podosomes. These structures were shown to be characterized by a lower turnover rate, compared to podosomes (half life time of 30–60 minutes in the case of large focal contacts, compared to 30 seconds to 5 minutes in podosomes). Strikingly, WIP^−/−^ cells treated with calpain inhibitors, in which WASp levels are restored, continue to exhibit these large focal contacts as their main adhesion structures; WASp rescue by calpain inhibitors does not induce podosome formation [18]. These findings emphasize the importance of WIP for the formation of podosomes.

Another role of WIP in the formation of podosomes was recently discovered in dendritic cells. The interaction between WIP and cortactin was found to be essential for podosome formation and maturation and for the recruitment of MMPs assisting in cellular migration by ECM degradation [45].

Phagocyte migration is dependent not only on the formation of podosomes, but also on their timely disassembly; the cycle of the assembly of podosomes, their recycling, and the formation of new podosomes propels the cell forward. It was suggested that calpain cleavage of WASp promotes podosome disassembly [37]. As WIP protects WASp from degradation, the regulation of WIP-WASp interaction may be an important factor in determining the fate of podosomes [19,90]. Further studies are required to clarify the role of WIP in podosome dynamics.

In mast cells, WIP was found to participate in degranulation and IL-6 secretion [91]. Following high affinity FCɛR ligation, the mast cell degranulation signal is transduced and propagated via Syk, a protein tyrosine kinase (PTK) involved in mast cell signaling and degranulation [92]. WIP serves as a chaperone of Syk, protecting it by preventing its degradation in the proteosomal pathway and possibly by interfering with calpain [91]. While the exact interaction site for WIP and Syk is yet to be mapped, it has been suggested [91] that this interaction is mediated by the CrkL adaptor protein and that a complex of WIP-CrkL-Syk is formed in activated mast cells similarly to the complex of WIP, CrkL and ZAP70 (a homologue of Syk) formed in activated T cells [42]. Additionally, WIP participates in actin polymerization and cytoskeletal rearrangement in mast cells, processes that are necessary for degranulation [91]. The multiple roles (both WASp dependent and WASp independent) are summarized in Figure 2.

## 5. Roles of WIP in Non-Immune Cells

In the past, most research regarding WIP activity and function was centered on cells belonging to the hematopoietic lineage. However, new studies have demonstrated that WIP plays important roles in multiple cell types. Given the cardinal role of WIP in the migration of leukocytes, it is not surprising that WIP was found to facilitate the chemotactic migration of fibroblasts [93–95]. Fibroblast mobility is a key activity in both development and wound healing. WIP overexpression results in impaired adhesion and spreading, while WIP^−/−^ cells exhibit increased spreading and adhesion. As both the formation and disassembly of adhesion structures are necessary for successful migration, these findings underscore the importance of WIP as a regulatory factor in cytoskeletal reorganization [95]. A study using the scratch assay demonstrated that migration in WIP overexpressing cells is diminished; however, while WIP^−/−^ cells were found to migrate more quickly than wild-type cells, another study, investigating chemotaxis with the use of Dunn chambers, showed that knockdown of WIP with siRNA disrupts the ability of fibroblasts to migrate towards an increasing concentration of PDGF. Additionally, PDGF uptake is reduced, although not abolished in these cells [94].

Interesting findings revealed the role of WIP as a negative regulator in neurons. Knockout of WIP was found to cause brain hypertrophy in mice, manifested in increased volume of the forebrain and the hippocampus. Neurons of WIP^−/−^ mice were found to exhibit increased development of neurites and neuronal branching, and increased synaptic maturation. While the mechanism by which WIP regulates neuronal development is currently unknown, it was suggested that WIP may participate in the regulation of cytoskeleton remodeling responsible for dendrite development and branching. By maintaining N-WASp in an auto-inhibitory conformation, WIP may restrain neurite development; in the absence of WIP, the development of neurites increases abnormally [96].

## 6. Roles of WIP in the Development and Pathology of Cancer

The process of metastasis includes the detachment of cancer cells from the primary tumor, and cellular invasion and migration into the connective tissue and blood vessels [97]. Cancer cell invasion during the process of metastasis requires the metastasizing tumor to penetrate through the ECM. Cancer cells invade the ECM by using actin rich structures called invadopodia [98].

Invadopodia are actin-based membrane protrusions consisting of actin filaments, regulatory proteins that control polymerization, signaling molecules, matrix proteinases, and adhesion molecules [87]. Invadopodia resemble podosomes in their appearance, their basic structure and their molecular composition. Moreover, studies have shown that podosomes are capable of ECM degradation [99]. This similarity has led to the suggestion that podosomes and invadopodia require similar proteins for their formation and biological function. It has also been shown that members of the WASp family, such as N-WASp and the WAVE family [100] are overexpressed in several cancer types [100,101]. RNA interference experiments have revealed that N-WASp is necessary for the formation of invadopodia; the requirement for the WIP-N-WASp interaction was found to be necessary for this process [102]. A model has been proposed, suggesting that formation of invadopodia is triggered by Epidermal growth factor (EGF) signaling, which results in the recruitment of the small GTPase of the Rho-subfamily, Cdc42, and of Nck. Nck then recruits the WIP-N-WASp complex to sites of invadopodium formation [102]. The precise role of WIP in invadopodium formation remains a subject for further research.

## 7. WIP Subverted—Intracellular Pathogens and Their Use of WIP

WIP, as well as other actin regulatory proteins such as Nck and N-WASp, is involved in microbial motility of certain intracellular pathogens. These pathogens, such as Shigella, Listeria, Mycobacteria and the Vaccinia virus, subvert the cellular actin machinery for their own use. These pathogens exploit the host’s actin polymerization machinery to migrate within host cells or to translocate from one cell to another, by generating formations termed “actin comets” or “actin tails” [103]. The viral-tail nucleator A36R protein of the Vaccinia virus was found to bind cellular WIP in order to recruit N-WASp and promote actin polymerization via the Arp2/3 complex. Vaccinia virions utilize these actin tails to spread from cell to cell [104]. Shigella, on the other hand, expresses the bacterial IcsA protein on its surface. IcsA binds to N-WASp, and through it, to WIP, thereby promoting actin polymerization, allowing these bacteria to migrate within host cells. Further understanding of these mechanisms may assist in the development of novel therapies directed at disrupting the exploitation by the pathogen of the actin polymerization machinery [105,106]. Increased understanding of the activity of WIP may further enable such research.

## 8. Concluding Remarks

Recent advances in the understanding of WIP functions and effector mechanisms have led to novel insights regarding the importance of WIP in the immune system and beyond. In addition to its known essential role as a WASp regulator and chaperone, WIP was revealed as a key regulator of multiple cellular processes (such as NK cell lytic granule polarization, outside-in integrin signaling in neutrophils via activation of the Hck PTK, and IL-2 responsiveness), as well as regulating actin polymerization and reorganization independently of WASp. Besides its critical roles in the immune system, WIP was found to participate in various processes in non-immune cells. Furthermore, WIP was found to play an important role in pathological processes. WIP is involved in cancer metastasis and invasion, as well as in the subversion of the cytoskeleton by certain intracellular pathogens.

Further investigation of WIP, its interactions with various binding partners, and the processes regulating its activity may provide a deeper understanding of the regulation and function of immune cells, and may pave the way for the development of novel therapies for primary immunodeficiencies, infectious diseases, and other pathological conditions. Studying the roles of WIP in cancer pathogenesis and in invasion of intracellular pathogens can also be of great importance for future clinical applications.
